# Computing in fish schools

**DOI:** 10.7554/eLife.12852

**Published:** 2016-01-18

**Authors:** Yaroslav Ispolatov

**Affiliations:** Departamento de Fisica, Universidad de Santiago de Chile, Santiago, Chilejaros007@gmail.com

**Keywords:** Collective behavior, Physical computation, Swarm, Optimization, Decision-making, Explore-exploit, None

## Abstract

A model based on shoaling fish suggests how a group can show decision-making properties beyond those of any one individual.

**Related research article** Hein AM, Rosenthal SB, Hagstrom GI, Berdahl A, Torney CJ, Couzin ID. 2015. The evolution of distributed sensing and collective computation in animal populations. *eLife* **4**:e10955. doi: 10.7554/eLife.10955

**Image** Social individuals (orange dots) can quickly find resource-rich areas (shaded area)

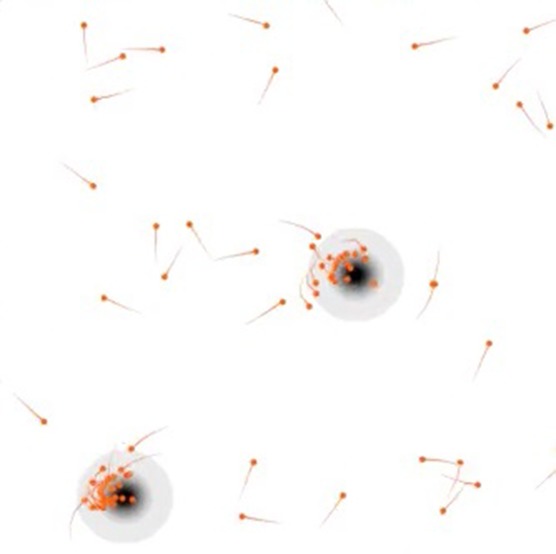

Many animals tend to move together as a group: swarms of insects, schools of fish, flocks of birds and herds of cattle. However, it is not known how and why the tendency to move as a group evolved, and the individual benefits conferred by such collective motion remain unclear. Now, in eLife, Andrew Hein, Iain Couzin and colleagues argue that a natural tendency to move in a group helps social individuals outcompete non-social members in the community (Hein et al., 2015). This is because abilities linked to collective motion help social individuals find resource-rich areas quickly.

Groups of animals can perform fairly complex collective behaviours. For example, a school of fish can quickly change its shape, density and direction of motion when being pursued by a predator or when finding somewhere to feed (Figure 1). One way to explain this collective motion is to make the fairly natural assumption that each individual monitors how close it is to its nearest neighbours and changes its speed and direction when such a neighbour gets too close to avoid collisions. However, if each individual is also attracted to more-distant neighbours, the group becomes capable of moving as a swarm and performing complex manoeuvres in a coordinated way (D’Orsogna et al., 2006, Herbert-Read et al., 2011).

**Figure 1.**
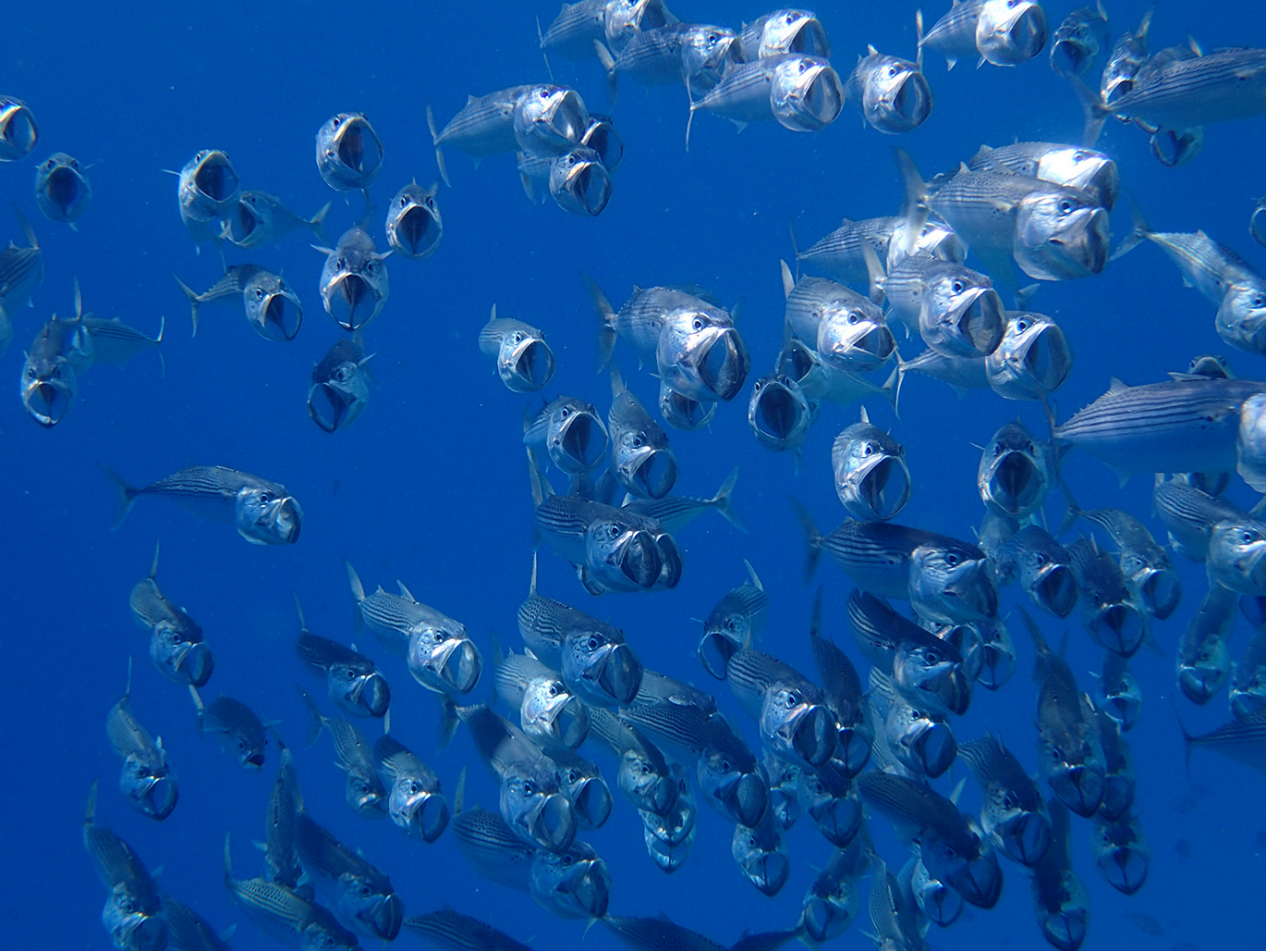
A shoal of fish in the Red Sea. How did collective motion, such as the movement of a school of fish, first evolve? And what do the individuals within the group gain from this collective behaviour? Hein et al. have developed a model based on individuals and groups searching for resources. This model reveals a number of emergent properties that allow groups to find resources more quickly than individuals working alone. Figure credit: Mike Kuznetsov

Importantly, collective motion does not require any individual to act as a leader, but instead results from individuals following a simple strategy: staying not too close and not too far from their neighbours. However, explaining how collective motion first evolved turns out to be more complex, partly because it touches on the controversial topic of how cooperation and altruistic behaviour evolved (Berdahl et al., 2013, Pratt et al., 2002, Torney et al., 2011).

To address this, Hein together with fellow joint first authors Sara Rosenthal and George Hagstom and other colleagues – who are based at Princeton University, the Max Planck Institute for Ornithology, the Santa Fe Institute, and the Universities of Exeter and Konstanz – developed a model of collective motion. Three parameters in this model were allowed to evolve: interaction range, cruising speed and slow-down rate (Hein et al., 2015). The ‘interaction range’ defines the furthest distance that an individual can be from one of its neighbours and still sense that neighbour. For short ranges, the interactions between individuals are dominated by reactions to avoid collisions. However, for longer interaction ranges, the attraction to individuals further away starts to play a role; this leads to cohesive groups as individuals are directed towards more crowded areas. ‘Cruising speed’ refers to how fast each individual travels when searching for resources, while the ‘slow-down rate’ refers to how quickly it slows down from this speed once it has found resources.

Resources appear and disappear at random in patches throughout the environment, and each individual in the model has a rather primitive ability to sense these resources. As such, the model describes fairly simple behaviours whereby each individual reacts to neighbours within a certain range and slows down in a resource-rich environment. The model is also set up such that individuals that spend more time in resource-rich patches produce more offspring; this allows more efficient foragers to outcompete the less efficient ones and take over the population.

This model revealed a universal trend: individuals evolve to essentially ‘crowdsource’ their senses and converge on resource-rich patches. Specifically, the interaction range evolves to be long enough to keep dense groups cohesive, while the cruising speed and the slow-down rate evolve to make exploration fast and efficient. Together the values of these three parameters evolve to enable a group foraging strategy. First, separate individuals scout the space until they encounter a resource-rich patch. This causes the individuals to slow down and stay longer inside the patch. Next, passing individuals sense this denser-than-average gathering of slow-moving individuals and are attracted to it. This allows the passing individuals to find patches of resources that they otherwise may have missed. Finally, once the patch is depleted, the group disperses back to scouting the wider environment.

Hein et al. point out that such a strategy depends on the interaction range evolving to be finely tuned to prevent the group swarming away from a patch too soon. They also show that such a strategy would be resistant to random mutations. This is because individuals who ignore their neighbours will tend to lose out to social individuals who are attracted to a feeding community without actually having to sense the resources themselves. Social individuals will thus spend less time than non-social individuals searching for resource-rich patches. Moreover, Hein at al. suggest that the evolved state is capable of more than collective resource sensing; that is, it can also perform ‘collective computation’. Indeed, as with the components of a computer, a group of individuals following simple rules can show complex sensing and decision-making properties that they are not capable of alone (so-called emergent properties).

In the future, a key challenge lies in testing this model and comparing its evolved parameters to ones inferred from experiments (Katz et al., 2011). Finally, the simplicity and intuitive appeal of the model put forward by Hein et al. means that it will likely be applicable to many biological systems found throughout nature.
